# A proof-of-concept sub-study exploring feasibility and preliminary evidence for the role of physical activity on neural activity during executive functioning tasks among young adults after cancer treatment

**DOI:** 10.1186/s12883-021-02280-y

**Published:** 2021-08-04

**Authors:** Amanda Wurz, Gladys Ayson, Andra M. Smith, Jennifer Brunet

**Affiliations:** 1 School of Human Kinetics, University of Ottawa, 125 University Private, Montpetit Hall, Room 339, K1N 6N5 Ottawa, Ontario Canada; 2 Present affiliation: Faculty of Kinesiology, University of Calgary, Alberta Calgary, Canada; 3 School of Psychology, University of Ottawa, Ottawa, Ontario Canada; 4 Cancer Therapeutic Program, Ottawa Hospital Research Institute, The Ottawa Hospital, Ottawa, Ontario Canada; 5 Institut du savoir Montfort, Hôpital Montfort, Ottawa, Ontario Canada

**Keywords:** Neuroimaging, fMRI, Brain function, Exercise, Cancer survivorship

## Abstract

**Background:**

Executive functioning (EF) deficits are troubling for adolescents and young adults (AYAs) after cancer treatment. Physical activity (PA) may enhance neural activity underlying EF among older adults affected by cancer. Establishing whether PA enhances neural activity among AYAs is warranted. As part of a two-arm, mixed-methods pilot randomized controlled trial (RCT), this proof-of-concept sub-study sought to answer the following questions: (1) is it feasible to use neuroimaging with EF tasks to assess neural activity changes following a 12-week PA intervention? And (2) is there preliminary evidence that a 12-week PA intervention enhances neural activity among AYAs after cancer treatment?

**Methods:**

AYAs in the pilot RCT were approached for enrollment into this sub-study. Those who were eligible and enrolled, completed functional magnetic resonance imaging (fMRI) with EF tasks (letter n-back, Go/No Go) pre- and post-PA intervention. Sub-study enrollment, adherence to scheduled fMRI scans, outliers, missing data, and EF task performance data were collected. Data were analyzed with descriptive statistics, blood oxygen level dependent (BOLD) analyses, and paired sample *t*-tests.

**Results:**

Nine eligible participants enrolled into this sub-study; six attended scheduled fMRI scans. One outlier was identified and was subsequently removed from the analytical sample. Participants showed no differences in EF task performance from pre- to post-PA intervention. Increases in neural activity in brain regions responsible for motor control, information encoding and processing, and decision-making were observed post-PA intervention (*p* < 0.05; *n* = 5).

**Conclusions:**

Findings  show that fMRI scans during EF tasks detected neural activity changes (as assessed by the BOLD signal) from pre- to post-PA intervention. Results thus suggest future trials confirming that PA enhances neural activity underlying EF are needed, though feasibility issues require careful consideration to ensure trial success.

**Trial registration:**

clinicaltrials.gov, NCT03016728. Registered January 11, 2017, clinicaltrials.gov/ct2/show/NCT03016728.

**Supplementary Information:**

The online version contains supplementary material available at 10.1186/s12883-021-02280-y.

## Background

Most adolescents and young adults (AYAs) diagnosed with cancer between the ages of 15–39 years survive the disease, though many are at increased risk for physical, psychological, social, and cognitive morbidity [1–3]. Cancer-related cognitive impairments, including executive functioning (EF) deficits, may be especially debilitating for AYAs after treatment. EF disturbances, characterized as perturbed mental skills needed for strategic planning, decision-making, reasoning, judgment, and self-monitoring, may impact AYAs’ educational and vocational pursuits after cancer treatment [4, 5]. This is because EF deficits make it difficult to focus, follow directions, and carry out plans independently and successfully [6]. Moreover, EF deficits can manifest as behavioral problems (e.g., poor impulse control, inability apprehending performance errors, difficulty making mental shifts), leading to difficulties in coping, completing tasks, handling emotions, and interacting with others [7, 8].

Despite this, EF is understudied among AYAs after cancer treatment [9]. Given the long-term EF deficits that have been described among older adults after treatment for breast cancer [10, 11], and the negative impact these deficits may have on quality of life [12], detecting EF changes among AYAs after treatment is important. In the few studies with AYAs diagnosed with cancer that have been published, researchers have used neuropsychological tests (i.e., performance-based methods) to assess EF [13]. Test scores may not fully capture subtle neurophysiological changes that occur after cancer treatment [14]. Pairing EF tasks, such as the letter n-back (capturing working memory; [15]) or the Go/No Go (capturing response inhibition; [16]), with neuroimaging techniques can be used to explore neural activity during EF task performance [17, 18]. Functional magnetic resonance imaging (fMRI) is one neuroimaging technique that can help detect differences in neural activity underlying EF, even in the absence of performance or detectable structural changes [19, 20]. It does so by using an indirect method of quantifying changes in blood flow via the blood oxygen level dependent (BOLD) signal, which is a proxy for neural activity [21]. When neural activity underlying EF is disrupted, which may occur during cancer treatment [22, 23], blood flow patterns would be expected to change. However, studies using fMRI during EF tasks to explore neural activity underlying EF among AYAs after cancer treatment are elusive.

Studies using fMRI with middle- and older-age adults who have completed treatment for breast cancer report underlying neurophysiological differences as indicated by changed neural activity, which may be related to worsened cognitive functioning, including EF [24]. For example, in one study, women who had completed treatment for breast cancer (*M*_age_=55.1.5±8.0 years) had lower prefrontal cortex activation during memory encoding and greater diffuse activation during verbal declarative memory recall tasks, when compared to healthy controls [25]. In another study with 60-year-old monozygotic twins, greater neural activity was seen in the twin who had undergone chemotherapy for breast cancer in the frontal and parietal regions of the brain during cognitive tasks that probe working memory [26]. Nevertheless, these results cannot be extrapolated to AYAs who have different cancer diagnoses and treatment trajectories, and who are transitioning through a period characterized by rapid brain reorganization and peaking cognitive abilities [27, 28]. To address this gap, studies exploring neural activity underlying EF using fMRI procedures among AYAs after cancer treatment are warranted.

In addition, intervening to mitigate EF deficits among AYAs after cancer treatment depends on identifying evidence-informed strategies. Physical activity (PA) may effectively mitigate EF disturbances. Indeed, there is evidence from experimental studies showing that PA may help to preserve cognitive functioning, including EF, among rodents treated with chemotherapy [29, 30]. Additionally, there is evidence from observational studies with middle- and older-aged adults that PA is positively related to EF after cancer treatment [31]. Further, data from experimental studies show benefits of PA on brain structure and neural activity underlying EF among adults over 50 years of age who completed a 12-week PA intervention after chemotherapy for lung cancer [32] and among women (*M*_age_=49.1±8.1 years) who completed a single 30-min bout of PA after treatment for breast cancer [33], respectively. Notwithstanding these contributions, the effects of PA on neural activity underlying AYAs’ EF after cancer treatment remains underexplored and unknown [34].

Whereas an adequately powered definitive randomized controlled trial (RCT) using neuroimaging with EF tasks is warranted to describe the effects of PA on AYAs’ neural activity after cancer treatment, a critical first step is to conduct a proof-of-concept study. Proof-of-concept studies are early-stage trials performed to explore whether an intervention (such as PA) is associated with specific outcomes (such as changes in neural activity underlying EF; e.g., [35–37]). Proof-of-concept studies are typically designed to include a fewer number of participants for a limited duration of follow-up. These studies are essential in helping decide whether to proceed with larger and more expensive phase III trials or to avoid expending resources on testing interventions that are not likely to succeed. Furthermore, a common objective of proof-of-concept studies are to detect a preliminary efficacy signal (e.g., a potentially valuable PA intervention effect), though they can also be useful to address a wide variety of other fundamental research objectives such as assessing safety and feasibility. They are therefore a valuable research tool in the identification of evidence-based interventions to mitigate cancer-related cognitive impairment among AYAs after cancer treatment.

### Current study

As part of a two-arm, mixed-methods pilot RCT, this proof-of-concept sub-study sought to answer the following questions: (1) is it feasible to use neuroimaging with EF tasks to assess neural activity changes following a PA intervention (as assessed via enrollment into the proof-of-concept sub-study, adherence to scheduled fMRI scans, outliers, missing data, and performance on EF tasks)? And (2) is there preliminary evidence that a 12-week PA intervention enhances neural activity among AYAs after cancer treatment?

## Methods

### Design

This study was a proof-of-concept sub-study, which took part within the context of a two-arm, mixed-methods pilot RCT designed to test the effects of a 12-week PA intervention on physical and psychological outcomes among AYAs after cancer treatment [38]. The protocol for the pilot RCT was registered in the ClinicalTrials.gov database (NCT03016728) and was approved by local Research Ethics Boards. The Consolidated Standards of Reporting Trials (CONSORT) 2010 checklist of information to include when reporting a pilot or feasibility trial [39] was adhered to in the preparation of this manuscript (see additional file 1).

### Participants

AYAs in the two-arm, mixed-methods pilot RCT were recruited through healthcare provider referral and self-referral over a 12-month period starting in September 2017. To participate in the pilot RCT, AYAs had to: (1) have been diagnosed with cancer between the ages of 15–39 years; (2) have completed cancer treatment within the past 5 years ; (3) have no evidence of progressive or recurrent disease or of secondary or second cancers; (4) be inactive/insufficiently active as assessed using a single-item screening question to which individuals had to respond negatively (*“Are you currently engaging in moderate PA, that is activity that increases your heart rate and causes you to sweat, more than three days/week?”*); (5) be medically cleared to participate in PA (as determined by a PA readiness questionnaire and in some cases a member of their healthcare team); and, (6) be able to read, understand, and provide informed consent in English. AYAs were not eligible if they self-reported having a physical impairment precluding participation in PA.

Following enrollment into the pilot RCT, participants who met the following criteria were invited to take part in this proof-of-concept sub-study: (1) self-reported being right-handed (e.g., writing and using a computer mouse with the right hand to increase the likelihood of recruiting a sample with left-language lateralization); (2) had no metal implants (e.g., pacemaker) or metal dental work (aside from fillings) that would preclude scanning; (3) were comfortable in small spaces (i.e., not claustrophobic); (4) had eyesight (correctable with contact lenses) that would enable them to view stimuli presented in the scanner; (5) would be able to lay relatively still for 1 h; and, (6) had not been diagnosed with a substance use disorder as assessed by a single-item screening question (*“Have you been told, in the last 5 years, by your healthcare provider that you have a substance use disorder?”*), to which they had to respond negatively. Nine out of the 16 participants enrolled into the pilot RCT were eligible and enrolled into this sub-study.

### Sample size

A power calculation was not performed given the objectives of this proof-of-concept sub-study. Rather, recruitment remained open and was tracked over a period of 12 months to assess the feasibility of year-round recruitment and data collection.

### Procedures

After providing written informed consent and being enrolled into the two-arm, mixed-methods pilot RCT by the first author, all participants completed a baseline assessment (week 0) at a location of their choosing (i.e., private room at the University of Ottawa, participants’ home, local cancer support organization) that included behavioral (PA behavior; assessed via self-report and accelerometry), physical (i.e., body composition, musculoskeletal strength, muscular endurance, resting blood pressure, aerobic capacity), and psychological (i.e., self-efficacy for PA, physical self-perceptions, physical self-esteem, global self-esteem) assessments and a qualitative interview. Once baseline assessments were completed, participants were informed by the first author whether they had been randomly assigned to the intervention group or the wait-list control group. Randomization was performed by an independent researcher using a random number generator (without an established allocation ratio) and sequentially labelled envelopes. All participants then completed a mid-intervention/waiting period assessment (week 6; behavioral, physical, and psychological assessments) and a post-intervention/waiting period assessment (week 12; behavioral, physical, and psychological assessments and a qualitative interview). Throughout the two-arm, mixed-methods pilot RCT, feasibility (i.e., recruitment metrics, retention, missing data) and adverse events were tracked (and are reported elsewhere; [38]). At study cessation, all participants were entered into a draw to win a $250 CAD gift card.

Participants who were eligible and enrolled into this proof-of-concept sub-study completed the above procedures, in addition to completing fMRI scans with EF tasks at the Royal Ottawa Mental Health Centre. fMRI scans were conducted concurrent with the baseline assessment (week 0), post-intervention/waiting period assessment (week 12), and 12-week post-intervention/waiting period assessment (week 24). Six of the nine participants enrolled into this sub-study completed all scheduled fMRI scans (i.e., adherence to the scheduled fMRI scans). In addition, enrollment into this proof-of-concept sub-study, outliers and missing data on sub-study assessments, and performance on EF tasks were tracked (see Results).

### Intervention group

Intervention group participants received a 12-week PA program, which was individualized using their baseline assessment results. Participants also received a yoga mat, water bottle, sweat towel, and socks (which they could keep) and were lent hand weights, resistance bands, and a Polar A300 monitor and heart rate strap (which they had to return post-intervention). Briefly, the 12-week PA intervention consisted of four weekly PA sessions, which lasted 25–45 min. The volume and intensity of each session was modified and progressed on an individual basis. Two sessions per week focused on strength activities (e.g., squats, lunges, shoulder presses) performed for 1–3 sets of 6–12 repetitions; these sessions were supervised by the first author^1^ for the first 6 weeks (at a location of participants’ choosing; i.e., private room at the University of Ottawa, participants’ home, local cancer support organization) and then were unsupervised for the remaining 6 weeks. Two sessions per week focused on aerobic activities (e.g., walking, rowing, indoor/outdoor bicycling, jogging) performed at 40–75 % of participants’ heart rate reserve. Aerobic sessions were unsupervised throughout. Participants were asked to self−monitor intensity using the Polar A300 monitor with a heart rate strap and/or a 10−point Perceived Exertion Scale. 

### Wait-list control group

Wait-list control group participants were asked to continue with their usual routine for 12 weeks. No restrictions were placed on their PA. After the 12-week intervention period, the wait-list control group participants received a 12-week individualized PA program in the same way as the intervention group.

### Data collection

As described in Procedures, multiple assessments were completed to collect data for the two-arm, mixed-methods pilot RCT. Henceforth, only measures and methods related to the objectives of this proof-of-concept sub-study are presented. Further details related to main pilot RCT objectives are published elsewhere [38].

### Sociodemographic, medical, and leisure time PA information

At baseline, participants self-reported their sex, age, age at cancer diagnosis, cancer type and treatments, education, and work status. In addition, they completed a modified version of the Leisure Time Exercise Questionnaire [40], wherein they reported the frequency and duration of leisure-time PA (i.e., PA performed during one’s free time) at mild (i.e., minimal effort; e.g., yoga, bowling, golf, easy walking), moderate (i.e., not exhausting; e.g., fast walking, tennis, easy bicycling, easy swimming), and vigorous intensities (i.e., heart beats rapidly; e.g., running, jogging, hockey, football). This information was collected to describe the sample.

### Feasibility

#### Enrollment to the proof-of-concept sub-study, adherence to scheduled fMRI scans, outliers, and missing data

To assess the feasibility of neuroimaging with EF tasks among AYAs, the number of participants from the pilot RCT who enrolled into this proof-of-concept sub-study and reasons for declining were recorded. As well, adherence to scheduled fMRI scans, outliers, and missing data on sub-study assessments were tracked.

#### EF task performance data

To examine whether the EF tasks worked as intended during the fMRI scanning sessions, participants’ performance (i.e., errors and reaction times) on EF tasks during the fMRI scans was documented.

### Preliminary evidence for the effect of PA on neural activity

Participants completed fMRI scans on a 3 Tesla Siemens Biograph Magnetom MR-PET scanner (Siemens, Erlangen, Germany) equipped with a 12-channel head coil. Whole brain echo planar fMRI was performed using a gradient echo pulse sequence (TR/TE 3000/34 ms, FA 90°, FOV 200 × 200 mm^2^, voxel size 1.6 mm × 1.6 mm × 3 mm, 48 axial slices, slice thickness 3 mm, band-width 2894 Hz). The total time for the scan was 1 h. At each assessment, the protocol was comprised of two EF fMRI tasks (described below; Letter n-back and Go/No Go), diffusion tensor imaging (DTI), and resting state fMRI. DTI and resting state fMRI results are presented elsewhere^2^.

### Letter n-back

During participants’ fMRI scan, a letter n-back task (designed by the third author) was presented. This task consisted of black letters presented in the middle of a white screen, one at a time, for 1500 milliseconds (ms) each with a 500 ms interstimulus interval (ISI). The block design task included two conditions: a control condition (‘Press for X’; a button press required for every X presented) and a working memory condition (‘Press for 2-back’; a button press required when the letter presented was the same as the one presented 2 letters prior). Instructions were presented for three sec before each block with ‘Press for X’ or ‘Press for 2-back’, respectively. There were no X’s presented in the ‘Press for 2-back’ blocks. Six blocks of each condition were performed with 16 stimuli presented randomly in each block. Six responses were required within each block. Rest periods were interspersed between blocks for 21 sec with the word ‘Rest’ on the screen.

#### Go/No Go

After the letter n-back task, while participants were still in the scanner, they completed the Go/No Go task (developed by the third author). The time between tasks was just enough to remind participants of the instructions for the Go/No Go task and to ensure they were comfortable continuing. The Go/No Go task consisted of black letters presented one at a time in the middle of a white screen for 75 ms with an ISI of 952 ms. Twelve stimuli were presented in each block, with four blocks of each condition: ‘Press for X’ (respond with button press for every X presented) and ‘Press for all letters except X’ (respond with button press when all letters other than the X were presented). Instructions were presented on the screen prior to each respective block for three sec and there were five required responses in each block. Fifty percent of the letters were X to build up a prepotent response to the X. Interspersed between the letter blocks were 21 sec rest periods with the word ‘Rest’ on the screen.

### Data processing and analysis

No formal hypothesis testing for efficacy was undertaken because the aim of this proof-of-concept sub-study was not to assess efficacy, and it was underpowered for this. Rather, descriptive statistics were computed to describe participants and to report on feasibility outcomes for the enrolled sub-study sample (*n* = 9) and the analytical sample (*n =* 5), using IBM SPSS 26 (IBM Corp.). Descriptive statistics included means with standard deviations and frequencies. EF task performance data were exported from E-Prime 2.0 [41] and were visually inspected to explore potential differences in performance (i.e., errors of commission or omission and reaction time for all correct responses occurring within 900 ms of stimulus presentation) from pre- to post-PA intervention on the letter n-back and Go/No Go. In addition, exploratory paired sample *t-*tests were performed to examine differences in performance from pre- to post-PA intervention. The fMRI data were post-processed and analyzed using Statistical Parametric Mapping (SPM) 12. The fMRI scans for both tasks were motion corrected through realignment, normalized to the standard SPM Montreal Neurological Institute template and spatially smoothed with an 8 mm FWHM Gaussian kernel. The letter n-back task images, for each person at each time point, underwent individual participant analyses with the ‘Press for 2-back’ minus ‘Press for X’ contrast as the working memory contrast of interest. Motion correction was applied as a regressor for all first-level analyses. Baseline fMRI scans (week 0) were treated as the ‘pre-PA intervention’ data for all participants as this was the first time participants saw the scanner and completed the EF tasks. The fMRI scans from week 12 and week 24 were treated as the ‘post-PA intervention’ data for the PA intervention group and wait-list group, respectively. A paired sample *t*-test comparing pre- and post-PA intervention fMRI scans during working memory processing and response inhibition tasks was conducted to address objective two and ascertain if there was preliminary evidence supporting the possible effect of PA on neural activity (as detected by the BOLD signal). The Go/No Go task images were analyzed in a similar procedure with the contrast of interest for response inhibition: ‘Press for all letters except X’ minus ‘Press for X’. All pre- and post-PA intervention analyses were whole brain investigations and were conducted at a set threshold of *p*_uncorr _= 0.001, with a cluster-wise correction at *p*_FWE_ = 0.05 and a set cluster size larger than 10 voxels.

As described above, proof-of-concept studies typically involve small sample sizes. However, this is at the expense of decreased statistical power and potential inability to detect statistically significant effects. Thus, proof-of-concepts studies may need to deviate from the standard significance level of 0.001. To this end, a higher type I error probability was set (i.e., an uncorrected significance level of 0.05) to decrease the risk of missing a potentially beneficial effect of PA.

## Results

### Feasibility

#### Enrollment to the proof-of-concept sub-study

**Fig. 1:**
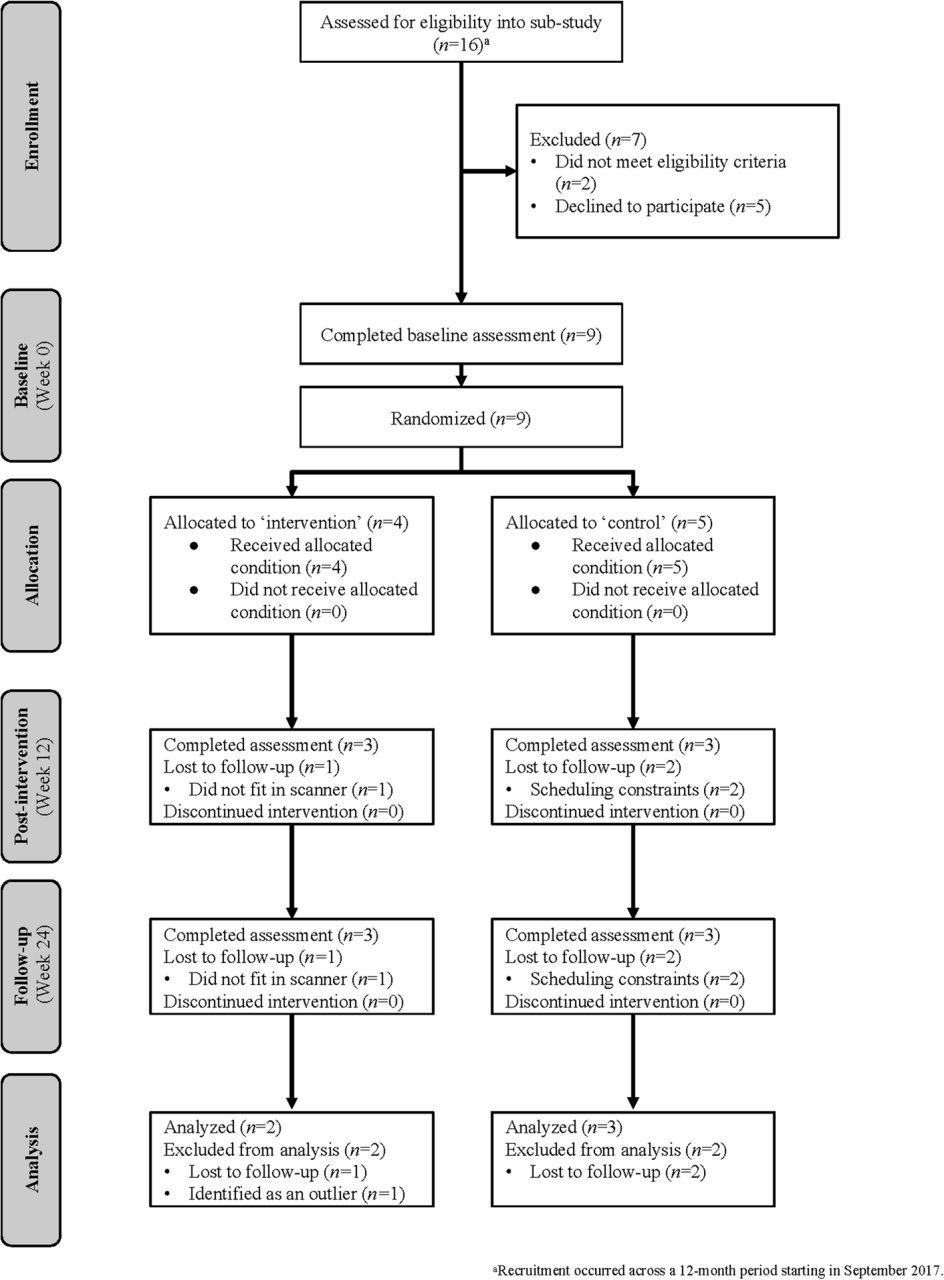
CONSORT flow diagram

All 16 participants from the two-arm, mixed-methods pilot RCT were approached to take part in this proof-of-concept sub-study. Five declined due to concerns of additional radiation, busy schedules, and/or number of imaging scans being completed as part of routine follow-up and two were ineligible (i.e., left handedness, metal implants). The remaining nine were eligible and enrolled into this proof-of-concept sub-study (Fig. 1). Although the sample size yields low power to detect differences between those enrolled and the remaining sample from the two-arm, mixed-methods pilot RCT, visual inspection of data suggest they were generally comparable at baseline in terms of key sociodemographics, medical information, and PA behavior, including proportion of participants self-identifying as female, educational attainment, household income, employment status, and type of cancer (Table 1). However, participants enrolled into this proof-of-concept sub-study appeared to be older than those who did not enroll at baseline and when diagnosed with cancer. Indeed, enrolled participants (*n* = 9) were on average 35.2 ± 5.6 years of age at baseline (compared to 29.8 ± 9.8 years for the seven who did not enroll) and 31.9 ± 5.9 years of age at diagnosis (compared to 26.7 ± 9.2 years for the seven who did not enroll). Four enrolled participants were randomized to the PA group and five to the wait-list control group. Most enrolled participants were female (*n* = 8; 89 %) and had been diagnosed with breast cancer (*n* = 5); the remaining had been diagnosed with biphasic peritoneal mesothelioma (*n* = 1), ovarian cancer (*n* = 1), rhabdomyosarcoma (*n* = 1), or soft tissue sarcoma (*n* = 1). On average, enrolled participants self-reported engaging in 52.8 (*SD* = 48.7) min of moderate-to-vigorous intensity PA per week at baseline. In turn, when looking at only those participants in the analytical sample (*n* = 5), they were on average 37.8 ± 2.0 years of age at baseline and were female (*n* = 5; 100 %), having been diagnosed with breast cancer (*n* = 3), ovarian cancer (*n* = 1), and soft tissue sarcoma (*n* = 1). Participants in the analytical sample self-reported engaging in an average of 20.0 ± 28.3 min of moderate-to-vigorous intensity PA per week at baseline.

**Table 1:** Baseline personal and medical characteristics of larger study and sub-study participants

	Two-arm, mixed-pilot RCT	Proof-of-concept sub-study	
	(*n* = 16)	Enrolled sample (*n* = 9)	Analytical sample (*n* = 5)^a^
Personal factors			
Female (*n*, %)	14 (87.5)	8 (88.9)	5 (100.0)
Age at baseline (mean years, *SD*)	32.8 (7.9)	35.2 (5.6)	37.8 (2.0)
Highest level of education (*n*, %)			
Some high school	2 (12.5)	1 (16.7)	1 (20.0)
Some university/college	4 (25.0)	2 (22.2)	--
Completed university/college	6 (37.5)	4 (44.4)	2 (40.0)
Some graduate school	1 (6.3)	--	--
Completed graduate school	3 (18.8)	2 (22.2)	2 (40.0)
Household income^b^ (*n*, %)			
Prefer not to answer	2 (12.5)	2 (22.2)	1 (20.0)
Do not know	2 (12.5)	--	--
< 20,000	1 (6.3)	1 (11.1)	--
20–39,999	2 (12.5)	--	--
40–59,999	--	--	--
60–79,999	1 (6.3)	1 (11.1)	1 (20.0)
80–89,999	--	--	--
90–99,999	--	--	--
> 100,000	8 (50.0)	5 (55.6)	3 (60.0)
Employment status^c^ (*n*, %)			
Disability	2 (12.5)	2 (22.2)	--
Student	4 (12.5)	--	--
Part-time employment	3 (18.8)	1 (11.1)	4 (80.0)
Full-time employment	8 (50.0)	6 (66.7)	1 (20.0)
Medical factors			
Age at diagnosis (mean years, *SD*)	29.6 (7.7)	31.9 (5.9)	34.8 (2.0)
Type of cancer (*n*, %)			
Breast	7 (43.8)	5 (55.6)	3 (60.0)
Biphasic peritoneal mesothelioma	1 (6.3)	1 (11.1)	--
Colorectal	1 (6.3)	--	--
Gastric	1 (6.3)	--	--
Hodgkin’s lymphoma	1 (6.3)	--	--
Osteosarcoma	1 (6.3)	--	--
Ovarian	2 (12.5)	1 (11.1)	1 (20.0)
Rhabdomyosarcoma	1 (6.3)	1 (11.1)	--
Soft tissue sarcoma	1 (6.3)	1 (11.1)	1 (20.0)
Time since treatment (mean years, *SD*)	2.2 (1.1)	2.4 (1.3)	1.9 (0.9)
PA behavior (mean, *SD*) Moderate-to-vigorous min/week	71.4 (102.6)	52.8 (48.7)	20.0 (28.3)

Notes. ^a^imaging analytic sample, accounting for loss to follow-up (*n* = 3) and outliers (*n* = 1); ^b^reported as Canadian dollars accrued per household annually, ^c^participants could select more than one option. *Min* minutes, *SD* standard deviation, *PA* physical activity. *RCT* randomized controlled trial

### Adherence to scheduled fMRI scans

All nine enrolled participants completed the baseline fMRI scan; however, three withdrew after the baseline fMRI due to scheduling constraints (*n* = 2) or not fitting inside the scanner (*n* = 1). Six participants (*n* = 3 intervention group; *n* = 3 waitlist control group) completed all scheduled fMRI scans (67 % adherence). See Fig. 1.

### Outliers and missing data

Of the six participants who completed all scheduled fMRI scans, one was identified as an outlier based on age at diagnosis, age at baseline, self-reported sex, and leisure-time PA. Because small sample sizes give individual participants significant influence on study outcomes, this participant was excluded from all data analysis and reporting to enhance homogeneity and maximize statistical power (though sensitivity testing was performed including this participant and similar results were observed). In addition, one participant did not have post-PA intervention EF task performance data available due to technical difficulties during their scan. Their imaging notes were consulted, and no EF task performance abnormalities were documented. As such, this participant’s EF task performance data were not available for visual nor statistical comparison pre- to post-PA intervention. Nevertheless, their notes and imaging results were retained to yield an analytical sample size of 5.

### EF task performance data

Participants’ EF task performance data revealed no notable differences in performance (i.e., errors of commission or omission and reaction time) from pre- to post-PA intervention based on visual inspection, wherein number of errors of commission (i.e., incorrect button press) and omission (i.e., no button press when a target stimuli is presented) and reaction time appeared similar pre- and post-PA intervention. Results from paired sample *t*-tests confirmed this, as there were no significant differences from pre- to post-PA intervention in total errors of commission on the letter n-back (*p* = 0.391) nor Go/No Go (*p* = 0.080). Additionally, there were no errors of omission at either time-point. Finally, there were no significant differences from pre- to post-PA intervention in reaction time on the letter n-back (‘Press for X’ condition, *p* = .109; ‘Press for 2-back’ condition, *p* = 0.317) and Go/No Go, (‘Press for X’ condition, *p* = 0.166; ‘Press for all letters except X’ condition, *p* = 0.072). Combined, these results indicate that the working memory (letter n-back) and response inhibition (Go/No Go) tasks worked as intended, since the tasks were designed to minimize performance differences. In addition, slower reaction times and more errors were observed for the ‘Press for 2-back’ condition compared to the ‘Press for X’ condition in the letter n-back task, which would be expected based on the working memory component of the ‘Press for 2-back’ condition, not present in the ‘Press for X’ condition. Similarly, slower reaction times and more errors during the ‘Press for all letters except X’ condition compared to the ‘Press for X’ condition were observed in the Go/No Go task, which was also expected based on increased reliance on response inhibition circuitry for withholding responding for the conditioned X during the former condition.

### Preliminary evidence for the effects of PA on neural activity

#### Letter n-back

**Fig. 2:**
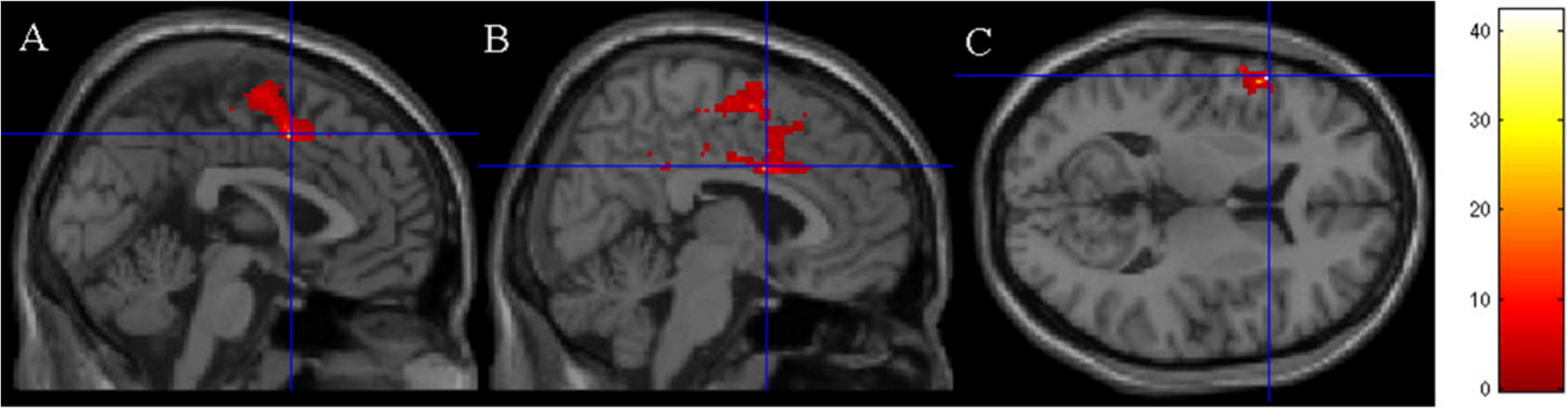
Results from the letter n-back task. Blue crosshairs are located on the most significantly different voxel of the (**A**) supplementary motor area, (**B**) middle cingulate gyrus, (C) frontal operculum. Color grid represents *t*-values

Significant increases in neural activity (as detected by the BOLD signal) were observed from pre- to post-PA intervention when completing the letter n-back task (see Fig. 2). The largest increases were seen in a cluster of 132,060 voxels and included the left inferior frontal operculum (x y z = -56 16 10, *t* = 42.29, *z* = 4.77, *p* = 0.000), supplementary motor area (x y z = 0 6 46, *t* = 25.83, *z* = 4.35, *p* = 0.000), the left precentral gyrus (x y z = -52 -4 38, *t* = 18.53, *z* = 4.05, *p* = 0.000), and the middle cingulate gyrus (x y z = 6 6 32, *t* = 21.4, *z* = 4.19, *p* = 0.000), which are responsible for working memory, planning complex movements, and cognitive control (see Discussion for references linking these areas of the brain to these functions).

#### Go/No Go

**Fig. 3:**
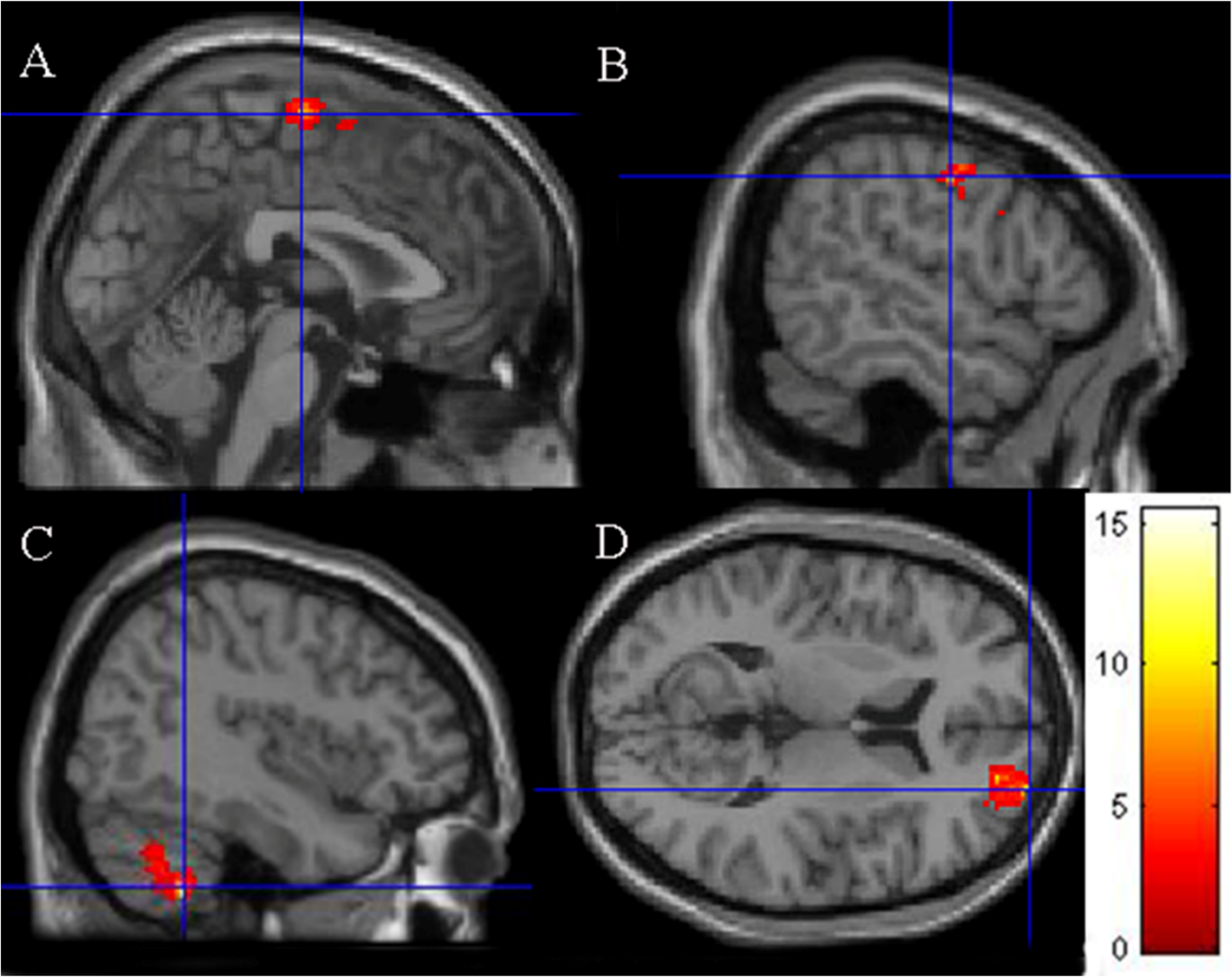
Results from the Go/No Go task. Blue crosshairs are located on the most significantly different voxel of the (**A**) supplementary motor area, (**B**) precentral gyrus, (**C**) superior frontal gyrus, (**D**) cerebellum. Color grid represents *t*-values

Significantly greater neural activity (as detected by the BOLD signal) was observed in several brain regions related to motor control, response inhibition and decision-making from pre- to post-PA intervention while completing the Go/No Go task (see Discussion for references linking these areas of the brain to these functions). Fig. 3 shows that the most significant increases were observed in two large clusters, including the right cerebellum (x y z = 20 -54 -36, *t* = 22.14, *z* = 4.22, *p* = 0.037, cluster size 1957 voxels), the supplementary motor area (x y z = 10 -12 68, *t* = 8.28, *z* = 3.25, *p* = 0.000, cluster size 4878 voxels), the precentral gyrus (x y z = 56 -8 42, *t* = 10.01, *z* = 3.45, *p* = 0.000, cluster size = 4878), and the right superior frontal gyrus (x y z = 26 66 10, *t* = 11.00, *z* = 3.55, *p* = 0.000, cluster size 4878 voxels).

## Discussion

EF deficits can be troubling for AYAs after cancer treatment as EF plays a critical role in many aspects of life and well-being. Generally, EF continues to develop and mature well into a person’s twenties, peaking during young adult years [28]. Yet, few efforts have been made to explore EF among young adults or AYAs after cancer treatment and little work has examined ways to improve EF in this population. As a first step, this proof-of-concept sub-study sought to answer two broad research questions. The first was to determine the feasibility of using neuroimaging with EF tasks among AYAs in terms of enrollment to the sub-study, adherence to scheduled fMRI scans, outliers, missing data, and EF task performance data. The authors acknowledge critical issues related to the feasibility of participant enrollment and adherence to scheduled fMRI scans. The second was to ascertain whether there was preliminary evidence to support further studies testing the effects of a 12-week PA intervention on AYAs’ neural activity underlying EF. Although it is necessary to take proof-of-concept studies for what they are – limited, small-scale studies addressing a focused research topic – the results provide a basis for future research. Interpretation of the results suggest that neural activity (as detected by the BOLD signal) may increase during EF tasks of working memory and response inhibition among young adults, who were not meeting PA guidelines at baseline, following a strength- and aerobic-based PA intervention. Specifically, findings suggest that PA may lead to greater neural activity in regions of the brain responsible for working memory, planning complex movements, cognitive control, motor control, response inhibition and decision-making [42–47]. Taken together, this study is an essential component of the exploratory development phase and results suggest it would be appropriate to embark on a larger study to assess the efficacy of PA for enhancing neural activity underlying EF among young adults after cancer treatment, provided feasibility issues are addressed. If shown to be efficacious, PA could be considered as a strategy to mitigate cancer-related changes seen in neural activity underlying EF, and perhaps more general cognitive functioning for this population.

Nearly one-third (5/16; 31 %) of participants approached to take part in this proof-of-concept sub-study declined, and two were ineligible (i.e., left handedness, metal implants). Reasons for declining related to fears of additional radiation (despite being assured that the fMRI scans did not pose a radiation threat) and the time required to complete these fMRI scans in addition to the other imaging scans they were completing as part of routine care. With regards to the former, fMRI is a non-invasive technology that does not include any ionizing radiation or chemical tracers. Researchers may wish to emphasize this with their prospective participants, provide them with resources they can review themselves, and/or consider having a healthcare provider (e.g., oncologist, nurse; who may be seen as more credible), deliver this information as these fears were a deterrent in this study. As for the barriers related to time and scheduling constraints, this was not surprising as other researchers have reported that time constraints are a major barrier to AYAs’ participation in clinical trials [48]. To enhance the success of future studies, exploring strategies to increase participant enrollment and ensure flexible assessment strategies are warranted.

Amongst those who were eligible and enrolled into this sub-study, one-third (3/9; 33 %) withdrew after the first fMRI scan. Reasons for withdrawing related to time and scanner constraints. With regards to the latter, one of the participants was unable to fit inside the scanner, and consequently withdrew. The ability to accommodate participants through the imaging equipment aperture is a necessary consideration. Considering the prevalence of obesity among AYAs after cancer treatment [49], scanner constraints are problematic and could result in samples that are not representative. In terms of outliers, one participant was identified as an outlier and was excluded from analyses. This was important because small sample sizes give each participant significant influence on study outcomes. Thus, to avoid having to discard collected data, appropriate participant selection is crucial. For trials evaluating preliminary efficacy, eligibility criteria should be formulated to yield a homogeneous sample to maximize statistical power. However, for trials designed to evaluate effectiveness, more heterogeneous samples may be required, and eligibility criteria could be broadened.

Notably, one participant had missing EF task performance data at their post-PA intervention scan due to technical difficulties. Missing data like this can be particularly problematic in small trials [50] because sample sizes and consequently power are already low. Depending on the nature and treatment of missing data, it is possible that estimates may be biased, leading to inaccurate conclusions. In an attempt to address this within the current sub-study, the missing EF task performance data were both visually inspected (enabling the inclusion of notes) and assessed via paired sample *t-*tests. Moving forward, accounting for technical difficulties and/or incorporating strategies to collect EF task performance data both via computer software and by hand may be warranted.

Taken together, the feasibility results from this proof-of-concept sub-study offer important considerations for researchers, such as the length of time that might be required to achieve desired sample sizes and amount of missing data. These results also underscore a crucial issue – that enrolled and adherent young adults may be highly motivated to participate in neuroimaging studies, and thus introduce self-selection, volunteer, or participation bias. As bias cannot likely be avoided, it will be important to ascertain the direction and, if possible, the magnitude of bias to adjust for it in future trials. Nevertheless, results also highlight that efforts to address these methodological challenges would be worthwhile as the fMRI with the letter n-back and Go/No Go tasks were sensitive to detect neural activity changes underlying EF among young adults after cancer treatment. This represents a major advancement for others wishing to assess neural activity among young adults (and AYAs) after cancer treatment.

Published findings show that PA may be a viable strategy to enhance neural activity underlying EF among middle- and older-aged adults after treatment for breast cancer [31, 33]. An objective of this study was to determine whether further research is needed to test this in AYAs after cancer treatment. This proof-of-concept sub-study provides information to aide in the decision to proceed with a larger, more comprehensive, and resource-intensive trial. Specifically, it provides initial evidence that researchers should continue to give attention to the potential effects of PA on neural activity underlying EF in this population. Moreover, it suggests that a 12-week PA intervention comprised of four weekly sessions of partially supervised strength activities and unsupervised aerobic activities may be an appropriate frequency, intensity, type, and length (time). This is not only indispensable to informing the design and development of PA interventions for AYAs to mitigate EF disturbances, but also provides early evidence to support stepped or triaged approaches (i.e., moving from supervised to unsupervised) if one is seeking more cost-effective interventions than fully supervised interventions; though efforts are still required to show efficacy and effectiveness.

Although this proof-of-concept sub-study advances the literature on PA and cognitive functioning, there are some limitations to take into account when interpreting the findings. First, given the nature of the larger pilot RCT, PA behavior during the waiting period was not assessed for waitlist control group participants, and thus it is not possible to know if they (intentionally or unintentionally) increased their PA behavior. Moving forward, researchers may wish to collect data on PA behavior concurrent with the intervention and fMRI scans, as well as collect more detailed PA behavior history (since long-term PA behavior may elicit changes in cognitive reserves; [51]) to control for PA behavior and its relationship with neural activity underlying EF. Relatedly, researchers may wish to add follow-up assessments to examine possible delayed or sustained effects of PA on neural activity underlying EF. Second, given the small sample size, between-group differences were not examined. Moreover, as with any study with a small sample size, conclusions are not generalizable to a larger population of AYAs. Indeed, the sample enrolled herein was comprised of a convenience sample of young adults from the two-arm, mixed-methods pilot RCT and the analytical sample self-identified as female. Given that various cognitive abilities peak throughout the lifespan [28] and that there is evidence for sex-specific differences [52] in factors underlying EF, exploring neurophysiological outcomes with samples comprised of older and younger male and female AYAs affected by cancer is required. There is a need to explore PA as a strategy for enhancing EF among AYAs with different diagnoses and who undergo disparate treatment regimens, and to consider potential confounding factors including sociodemographic (e.g., age, education) and medical variables (e.g., cancer type, treatment duration) in future work. Third, due to the nature of the two-arm, mixed-methods pilot RCT design, there were differences in the length of time between the pre- and post-PA intervention fMRI scans for participants in the intervention group versus the wait-list group, which could have impacted results. It was thought, however, that using the first scan as a baseline for both groups was more appropriate than using the second scan for the wait-list group, so as to avoid practice effects. Finally, this study did not include long-term follow-up assessments as it is unknown how long it may take for neural activity to change with PA. As such, whether enhanced neural activity was maintained could not be examined.

## Conclusions

This proof-of-concept study provides a basis for future research. It provides feasibility data to guide the development of future definitive RCTs. Specifically, it suggests efforts to improve enrollment rates and enhance protocol adherence may improve the efficiency of future trials. Additionally, it supports embarking on a larger trial as the data obtained from the fMRI scans with the letter n-back and Go/No Go tasks suggest that PA may affect neural activity underlying EF among AYAs after cancer treatment. Accordingly, it provides a strong basis for future research and new opportunities to mitigate cancer-related cognitive impairment, including EF deficits, that interfere with AYAs’ lives and potentially harm their overall well-being.

## Supplementary Information

**Additional file 1.**

## Data Availability

The data cannot be shared as participants were assured that their data would be kept private and confidential to the extent permitted by law, and that only the research team would have access to the data. However, summarized and de-identified data can be available at reasonable request to the corresponding author.

## Abbreviations

AYA: Adolescent and young adult
BOLD: Blood oxygen level dependent
CONSORT: Consolidated Standards of Reporting Trials
DTI: Diffusion tensor imaging
EF: Executive functioning
fMRI: Functional magnetic resonance imaging
ISI: Interstimulus interval
ms: Millisecond
PA: Physical activity
RCT: Randomized controlled trial
SPM: Statistical Parametric Mapping

## Footnotes

1 The first author is a certified personal trainer with experience leading group-based and one-on-one PA for individuals affected by cancer.

2 See Lambert M, Wurz A, Smith AM, Zhuo F, Brunet J. Preliminary evidence of improvement in adolescent and young adult cancer survivors’ brain health following physical activity: a proof-of-concept sub-study. Brain Plast. 2021; pre-press:1-13; doi: 10.3233/BPL-210124.

## Declarations

### Dual publication

The authors note that the sub-study presented in the current manuscript is part of a larger pilot randomized controlled trial. Although additional manuscripts using the dataset from the pilot randomized controlled trial have been, and will be, published, these manuscripts ask different research questions and/or utilize different questionnaires and methods to assess constructs unrelated to what is presented herein. As such, the overlap is very minimal, and the authors are confident that the current manuscript is uniquely informative and independent of these contributions.
